# Acute stress modulates hippocampal to entorhinal cortex communication

**DOI:** 10.3389/fncel.2023.1327909

**Published:** 2023-12-07

**Authors:** Azat Nasretdinov, David Jappy, Alina Vazetdinova, Fliza Valiullina-Rakhmatullina, Andrei Rozov

**Affiliations:** ^1^Laboratory of Neurobiology, Kazan Federal University, Kazan, Russia; ^2^Federal Center of Brain Research and Neurotechnologies, Moscow, Russia; ^3^Institute of Neuroscience, Lobachevsky State University of Nizhny Novgorod, Nizhny Novgorod, Russia; ^4^Department of Physiology and Pathophysiology, Heidelberg University, Heidelberg, Germany

**Keywords:** feed-forward inhibition, CB1R, SPW-R, stress, endocannabinoids

## Abstract

Feed-forward inhibition is vital in the transfer and processing of synaptic information within the hippocampal–entorhinal loop by controlling the strength and direction of excitation flow between different neuronal populations and individual neurons. While the cellular targets in the hippocampus that receive excitatory inputs from the entorhinal cortex have been well studied, and the role of feedforward inhibitory neurons has been attributed to neurogliafom cells, the cortical interneurons providing feed-forward control over receiving layer V in the entorhinal cortex remain unknown. We used sharp-wave ripple oscillations as a natural excitatory stimulus of the entorhinal cortex, driven by the hippocampus, to study the function of synaptic interactions between neurons in the deep layers of the entorhinal cortex. We discovered that CB1R-expressing interneurons in the deep layers of the entorhinal cortex constitute the major relay station that translates hippocampal excitation into efficient inhibition of cortical pyramidal cells. The impact of inhibition provided by these interneurons is under strong endocannabinoid control and can be drastically reduced either by enhanced activity of postsynaptic targets or by stress-induced elevation of cannabinoids.

## Introduction

The hippocampal-entorhinal loop plays an important role in episodic memory, storing spatial and temporal information about the occurrence of past events. Over the past decade, significant progress has been made in understanding the function and postsynaptic targets of projections from the entorhinal cortex (EC) to the hippocampus (Zhang et al., 2013, 2014; Kitamura et al., 2015; López-Madrona and Canals, 2021). However, until recently, little was known about projection pattern of the hippocampus to the EC (Sürmeli et al., 2015; Witter et al., 2017). Moreover, while local excitatory/inhibitory circuitries in the hippocampus (Freund and Buzsáki, 1996; Freund and Katona, 2007) and upper layers of EC (Varga et al., 2010; Armstrong et al., 2016; Witter et al., 2017) have been investigated and specific functional roles assigned to given types of interneurons, nearly nothing is known about feed-back and feed-forward inhibition in the deep layers of EC.

**FIGURE 1:**
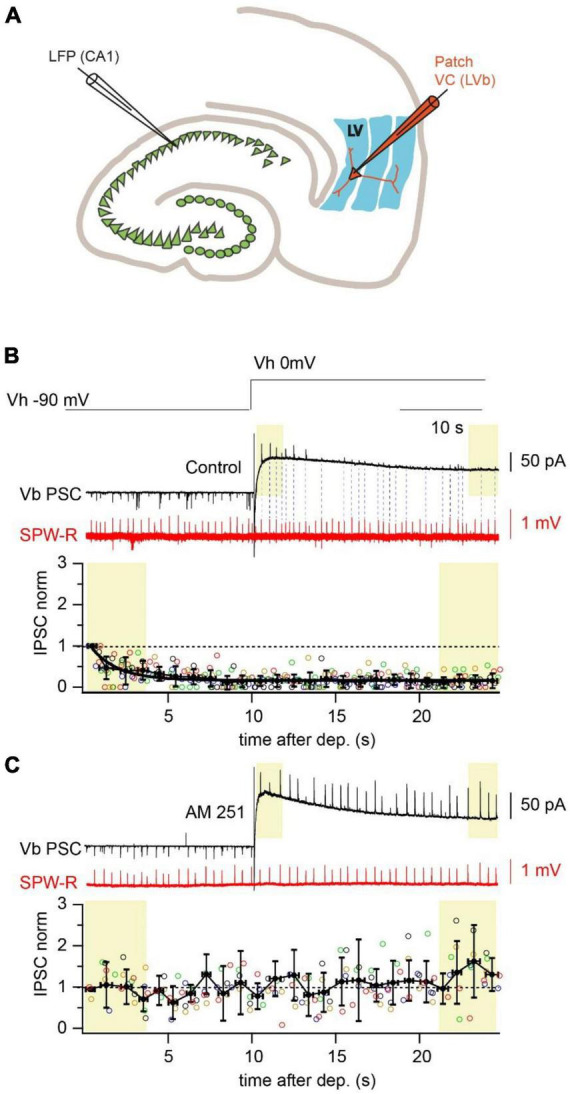
Depolarization reduces amplitude and propagation probability of SPW-R driven IPSCs. **(A)** Schematic representation of a horizontal hippocampal–EC slice with the position of the LFP recording site in the CA1 region and a LVb pyramidal neuron in layer V of EC recorded in whole cell voltage-clamp mode. **(B)** Spontaneous SPW-Rs (red) in CA1 and associated PSCs (black) in mEC LVb recorded at the reversal potentials of GABA_A_ (–90 mV) and iGluR (0 mV). Experiments were done with a low-Cl^–^ internal solution. Note that right after the depolarization step IPSCs are well detectable. However, both IPSC amplitude and propagation probability rapidly decline at the depolarized holding potential. Dotted lines connect SPW-Rs with corresponding small amplitude IPSCs. The time course of depolarization induced reduction of IPSC amplitudes is plotted underneath. Data from different neurons are shown in different colors (*n* = 5; *p* < 0.01). For statistical analysis the IPSC amplitudes within the first and the last 3 s windows (yellow boxes) after depolarization were compared. **(C)** The same as in panel **(A)** recorded in the presence of a CB1R blocker (AM251; 2 μM). Application of the CB1R antagonist completely occludes the effect of depolarization on IPSC amplitude (*n* = 5; *p* > 0.05).

In our previous study we characterized the functional connectivity between the ventral hippocampus and the deep layers of the medial entorhinal cortex (mEC) (Rozov et al., 2020). Besides direct projections to two types of layer V pyramidal cells, we discovered that deep layer fast-spiking interneurons (FS-IN) also receive hippocampal excitatory inputs. It was suggested that during rhythmic activity FS-IN can be recruited into hippocampal-driven feed-forward inhibition. Indeed, during sharp-wave ripples (SPW-R) we observed IPSCs in LVa and LVb pyramidal neurons (held at 0 mV) with a characteristic disynaptic delay relative to the onset of hippocampal SPW-R. However, IPSC-coupling, calculated as the percentage of SPW-R followed by IPSCs in both types of pyramidal cells was significantly lower than EPSC-coupling when measured from the same cell (Rozov et al., 2020). A close look at the coupling and amplitude dynamics of SPW-R driven IPSCs revealed that right after depolarization nearly every SPW-R event was followed by a high amplitude IPSC, but then both amplitude and coupling probability drastically declined reaching steady state values within 30 s. In the present study we found that suppression of SPW-R associated IPSC amplitudes and coupling probabilities were occluded by application of the CB1 receptor antagonist AM-251 (2 μM), suggesting involvement of CB1-positive interneurons in SPW-R driven feed-forward inhibition (Gatley et al., 1996; Figure 1). Thus, one can assume the following: (i) depolarization of the postsynaptic neuron triggered synthesis of endocannabinoids, which selectively blocked GABA release from CB1R expressing terminals; (ii) CB1R-positive interneurons (CB1-IN) also receive direct hippocampal innervation and are involved in signal processing within the hippocampal-entorhinal loop.

Interestingly, differential roles of CB1-INs and FS-INs in the control of neuronal populations involved in propagation of information within the hippocampal-entorhinal loop have been previously suggested for both the hippocampus (Yap et al., 2021) and upper layers of EC (Witter et al., 2017). Expression of CB1R equips the circuitries controlled by CB1-INs with the possibility of activity dependent modulation of the inhibitory impact of these interneurons (Dubruc et al., 2013). Moreover, the strength of CB1-IN - mediated inhibition can be altered by circulating endocannabinoids, and the concentration of the latter can be elevated by salient aversive experiences and acute stress (Ney et al., 2021; Vecchiarelli et al., 2022; Kondev et al., 2023a,b).

Therefore, in this study we explore: (i) the integration of the deep layer EC CB1INs in long distance (hippocampal-entorhinal loop) and local (layer V) networks; and (ii) the impact of stress-dependent endocannabinoid modulation in feed-forward CB1IN-mediated inhibition.

## Results

### Synaptic integration of CB1R-positive interneurons into the layer V mEC network

**FIGURE 2:**
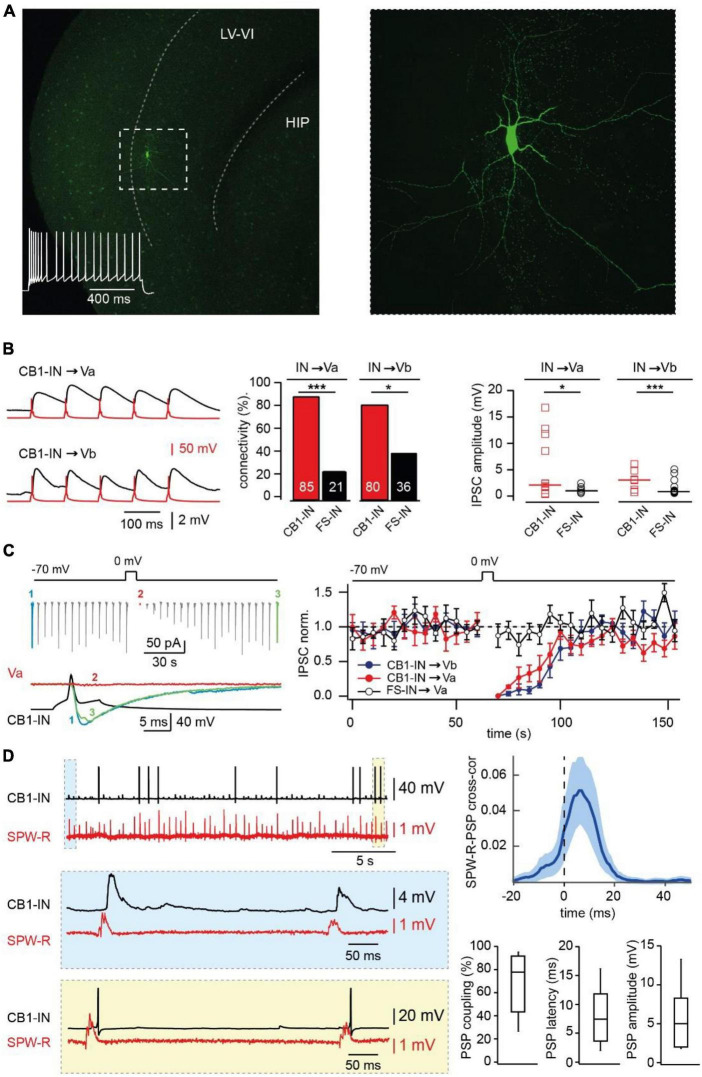
Synaptic integration of deep layer entorhinal cortex CB1R positive interneurons into local and long-distance networks. **(A)** z-Projected confocal images of a biocytin-filled mEC layer V CB1-IN at low (left) and high (right) magnification. The characteristic firing pattern is shown on the left panel. **(B)** Connectivity and efficacy of inhibitory connections from CB1-INs to LV pyramidal neurons in comparison with the properties of corresponding FS-INs connections. The left panel shows example traces recorded from connected CB1-IN to Va and Vb pyramidal neurons. The bar histogram (center) compares connectivity from two types of mEC LV interneurons to local pyramidal cells. The number of trials for different types of connections are as follows: CB1-IN to Va (*n* = 20), CB1-IN to Vb (*n* = 20), FS-IN to Va (*n* = 29) and FS-IN to Vb (*n* = 27). The significance of the differences was assessed by Fisher’s exact test (*p < 0.05; ***p < 0.001). The right plot compares individual amplitudes of IPSPs and median values at: CB1-IN to Va (*n* = 12), CB1-IN to Vb (*n* = 7), FSIN to Va (*n* = 6) and FSIN to Vb (*n* = 15) connections. The significance of the differences was assessed by Mann-Whitney test (*p < 0.05; ***p < 0.001). For calculating amplitude distribution, we used only those experiments which were done in CC mode. For calculating connectivity, we used both experiments done in VC and CC modes. Data on connectivity rate and synaptic efficacy at connections formed by FS-INs were taken from Rozov et al. (2020). **(C)** DSI at CB1-IN to Va and Vb cell synapses. An example experiment is shown on the left. The plot on the right compares depolarization-induced changes in synaptic efficacy at the following synapses: red–CB1-IN to Va (*n* = 5; *p* < 0.001), blue - CB1-IN to Vb (*n* = 5; *p* < 0.001) and black–FS-IN to Va (*n* = 5; *p* > 0.05) pyramidal cells. For statistical analysis the IPSC amplitudes within the 10 s windows prior to depolarization and right after depolarization were compared. **(D)** Propagation of SPW-Rs to mEC LV CB1-INs. PSPs of mEC CB1-IN (red trace) during spontaneous SPW-Rs in CA1 (black trace). Expanded traces of subthreshold SPW-R driven PSPs (blue box) and SPW-R driven APs (yellow box) are shown underneath. Averaged cross-correlograms between SPW-Rs and PSPs in CB1-INs (upper right plot; peak values: mean ± SD; *n* = 8). **(C)** Box plots show pooled data on PSP coupling, PSP latency, and PSP amplitude for SPW-R driven responses (*n* = 8). Data are presented as the median (P25; P75).

Screening the different interneurons located in layer V of mEC we found a population of cells that have firing properties similar to those described for CB1R-positive hippocampal basket cells (Pawelzik et al., 2002). In response to a 1 s depolarizing current injection these interneurons fire action potentials with a characteristic initial burst followed by lower frequency regular spiking (Figure 2A). The neurons were located in layer Va, the size and the appearance of the cell body on the IR-image was similar to that of Va pyramidal cells. Therefore, all experiments that required direct recordings from CB1R-expressing interneurons were performed in slices from GAD67-GFP mice.

To confirm expression of presynaptic CB1Rs in these neurons we tested for the ability to exhibit depolarization-induced suppression of inhibition (Kreitzer and Regehr, 2001; Wilson et al., 2001) (DSI) at their synapse onto local pyramidal LVa and LVb cells. The connectivity of the interneurons to both types of LV pyramidal cells was very high, being 85% (17 out of 20 tested pairs) for connection to LVa pyramids and 80% (16 out of 20 tested pairs) for connection to LVb pyramids (Figure 2B). In all tested cases depolarization of the postsynaptic pyramidal cell caused strong DSI lasting for more than 30 s (Figure 2C), confirming CB1R expression in these interneurons (CB1-IN). Another feature that was similar between hippocampal and mEC CB1-INs was profound asynchronous GABA release in response to high frequency stimulation (Hefft and Jonas, 2005; Ali and Todorova, 2010; Supplementary Figure 1A).

### Involvement of CB1R-positive interneurons in SPW-R driven feed-forward inhibition

**FIGURE 3:**
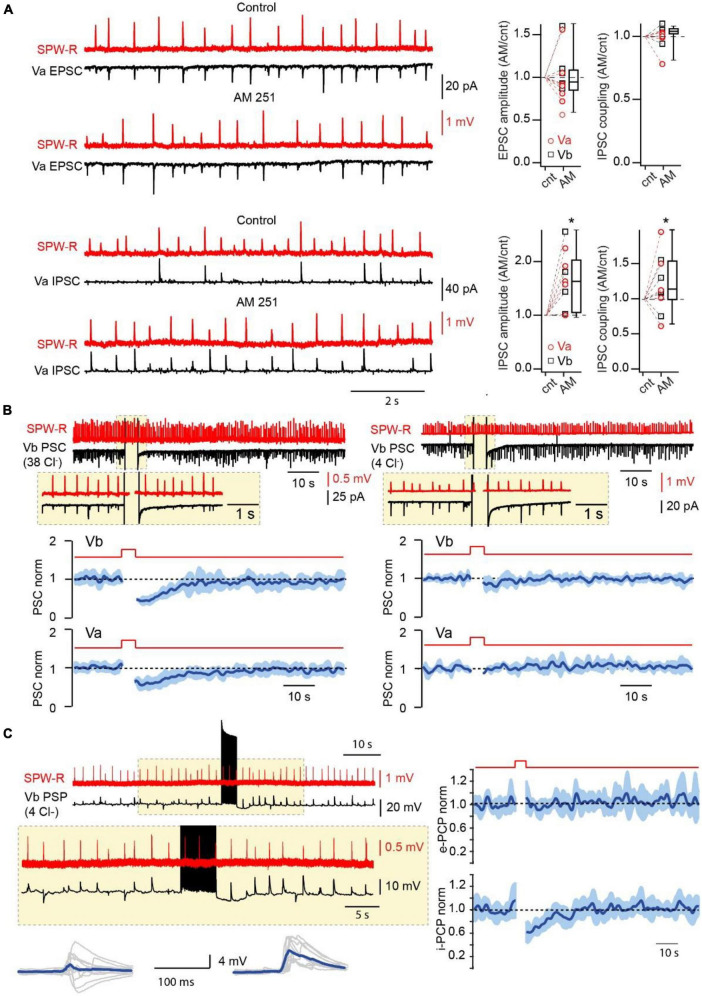
Endocannabinoids control the amplitude and probability of SPW-R driven inhibition. **(A)** Effect of CB1R blockade on the amplitude and coupling probability of SPW-R driven EPSCs and IPSCs. Traces on the left show spontaneous SPW-Rs in CA1 and associated EPSC (V_h_ –90 mV) and IPSC (V_h_ 0 mV) in mEC LVa cells before and after application of the CB1R antagonist AM251 (2 μM). The experiment was done with a low-Cl^–^ internal solution. Box plots on the right show normalized (AM251/control) amplitude and coupling probability values in individual cells and pooled data (presented as the median; P25; P75). The significance of the differences was assessed by Wilcoxon Signed Rank Test (*p < 0.05). Note, that application of AM251 did not have any effect on EPSCs but caused significant enhancement of the amplitude and coupling probability of IPSCs. **(B)** Effects of short-lasting (5 s) depolarization on the amplitude of compound SPW-R driven PSCs depends on internal Cl^–^ concentration. Traces on the left represent spontaneous SPW-Rs (red) in CA1 and associated PSC (Vm –90 mV) in mEC LVb recorded with a high-Cl^–^ (38 mM) internal solution. Depolarization of the postsynaptic cell led to temporal reduction of PSC amplitudes. Plots underneath show averaged time course (mean ± SEM) of induced by depolarization suppression of PSC efficacy in Va (*n* = 7; *p* < 0.01) and Vb (*n* = 7; *p* < 0.01) pyramidal cells. The traces and the plots on the right represent data obtained with a low-Cl^–^ (4 mM) internal solution. Each plot represents data from 7 cells (*p* > 0.05). For statistical analysis the PSC amplitudes within the 5 s windows prior to depolarization and right after depolarization were compared. **(C)** Postsynaptic cell burst firing triggers a DSI-like effect. Traces on the left represent spontaneous SPW-Rs (red) in CA1 and associated PSPs (black; Vm –65 mV) in mEC LVb (*n* = 7) recorded with a low-Cl^–^ internal solution. A train (5 s) of high frequency APs in the postsynaptic cell temporally enhances amplitude and duration of PSPs. Traces underneath show 10 individual PSPs (gray) and averaged responses (blue) before and after the AP burst. Plots on the right show averages of normalized amplitudes of excitatory (e-PSP; *p* > 0.05) and inhibitory (i-PSP; *p* < 0.01) components of SPW-R driven responses. For statistical analysis the e-PSP and i-PSP amplitudes within the 5 s windows prior to depolarization and right after depolarization were compared.

Next, we assessed whether CB1-INs in mEC LV receive direct excitatory drive during SPW-Rs. Oscillatory activity was measured extracellularly in the CA1 region of the hippocampus simultaneously with whole cell current clamp recordings of cortical CB1-INs identified by firing properties (Hájos and Mody, 2009; Rozov et al., 2020; Figure 2D). In all cases (*n* = 8) hippocampal SPW-R could trigger subthreshold responses in interneurons with characteristic propagation success and latencies that were very similar to those found for mEC FS-Ins (Rozov et al., 2020). Moreover, in 37.5% of recorded CB1-INs, SPW-R driven responses could reach suprathreshold values and trigger action potentials (APs). The high excitability of CB1-INs (Supplementary Figure 2) together with their high connectivity rate to layer V mEC pyramidal neurons strongly suggest that these interneurons play a significant role in feed-forward inhibition, which was overlooked in our previous study, since the depolarization used for IPSC separation leads to DSI and selectively occluded the contribution of CB1-INs. To test this hypothesis, we evaluated the effects of CB1R blockade on amplitude and coupling probability of SPW-R driven EPSCs and IPSCs. As expected, application of AM251 did not affect EPSCs, while both amplitude and coupling frequency of IPSCs recorded at 0 mV in LVa and LVb pyramidal cells after drug application were substantially higher relative to control (Figure 3A).

Thus, CB1-INs are indeed among the major players in hippocampal feed-forward inhibition of layer V mEC. Moreover, given that we didn’t find any excitatory connections from LVb pyramidal cells to CB1-IN (*n* = 20) and only one connection from LVa (*n* = 20) to CB1-INs, hippocampal glutamatergic projections might constitute the main source of excitatory drive to CB1-INs, which makes the role of these interneurons within the hippocampal-mEC loop significant.

### Endocannabinoid modulation of CB1-IN feed-forward inhibition

To assess the possible specific function of CB1-INs in signal transduction between these two structures we first tested if depolarization can reduce the contribution of CB1-INs to SPW-R driven IPSCs. This task was rather challenging since pharmacological isolation of IPSCs by blocking excitation would also block rhythmic activity (Maier et al., 2003). To overcome that problem, we used a high Cl^–^ internal solution (38 mM). In this case E_*GABA*_ is around −40 mV, while at −90 mV the direction of IPSCs and EPSCs is the same. Thus, DSI selectively reducing IPSCs would also suppress the amplitude of compound SPW-R driven responses (PSC) recorded at −90 mV. Indeed, 5 s depolarization of postsynaptic LVa (*n* = 7) and LVb (*n* = 7) pyramidal cells to 0 mV led to significant temporal reduction of SPW-R driven PSC amplitudes (Figure 3B). The DSI-like effect was totally occluded by application of the CB1 antagonist AM251 (2 μM; Supplementary Figure 3). To exclude possible effects of depolarization on the excitatory component of PSCs we repeated the same experiments with a low Cl^–^ internal solution (4 mM; E_*GABA*_ ∼−90 mV). Under these conditions depolarization didn’t have any significant effect on PSCs recorded at −90 mV (Figure 2B; *n* = 7 for both LVa and LVb neurons). We then tested whether endocannabinoids could modulate the amplitude and duration of SPW-R driven PSPs under more physiological conditions, namely: in current clamp mode with a low intracellular Cl^–^ concentration (4 mM) and the use of burst firing (Dubruc et al., 2013) instead of sustained depolarization to trigger DSI. These experiments were done on LVb pyramidal cells, since they have shorter afterhyperpolarization and recover back to resting membrane potential faster than LVa pyramidal neurons. Prior to firing activity most PSPs had prominent depolarizing and hyperpolarizing components, however, within 10–20 s of postsynaptic burst firing, the contribution of the GABA-mediated inhibitory component was significantly reduced, which led to enhancement of the amplitude and duration of PSPs (Figure 3C). Application of the CB1R antagonist completely occluded the DSI-like effect of postsynaptic cell firing on SPW-R driven IPSP (Supplementary Figure 4) We separately analyzed the excitatory and inhibitory components of PSPs (Antoine et al., 2019) (for details see method; Supplementary Figure 5). The time course of suppression of inhibition was very similar to the durations of DSIs observed for IPSCs in connected cell pairs and for SPW-R driven PSCs. Thus, endocannabinoid-dependent suppression of CB1-IN-mediated feed-forward inhibition should be more pronounced in pyramidal cells that receive stronger excitatory inputs, therefore, promoting further firing activity in more active neurons.

### Acute stress reduces the impact of CB1-IN-mediated feed-forward inhibition via activation of presynaptic CB1Rs

**FIGURE 4:**
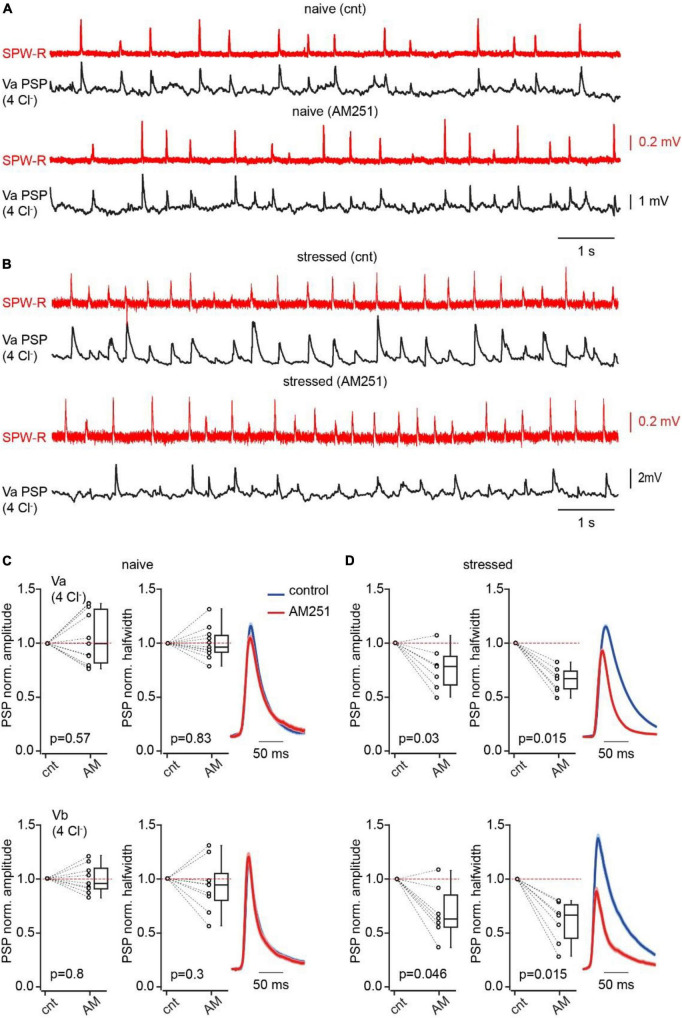
Acute stress suppresses CB1IN mediated feed-forward inhibition. **(A)** Spontaneous SPW-Rs (red) in CA1 and associated PSPs (black; Vm –65 mV) in mEC LVa recorded in a slice from a naive animal before and after AM251 application. **(B)** The same as in panel **(A)** recorded in the brain slice of the mouse that was stressed prior to decapitation. **(C)** Plots show normalized (AM251/control) amplitude (left) and halfwidth (right) values in individual cells and pooled data (presented as the median; P25; P75) obtained in Va (upper plots; *n* = 11) and Vb (bottom plots; *n* = 9) pyramidal cells, recorded in brain slices of naïve mice. **(D)** The same as in panel **(A)** recorded in brain slices of stressed mice (Va *n* = 7; Vb *n* = 7). Note that in both types of pyramidal neurons blockade of CB1R results in significant reduction of PSP amplitude and duration in slices obtained from stressed mice. The significance of the differences was assessed by the Wilcoxon signed-rank test, p values are indicated on the plots.

In the DSI-like phenomenon, endocannabinoid synthesis is triggered by activity of individual neurons, however, certain levels of endocannabinoids are persistently present in the brain tissue causing detectable suppression of GABA release via partial activation of CB1Rs (Neu et al., 2007; Ali and Todorova, 2010; Lee et al., 2010). The concentration of circulating endocannabinoids can increase in response to various adverse stimuli for example: acute stress (Ney et al., 2021; Vecchiarelli et al., 2022; Kondev et al., 2023a,b), tissue injury (Vered et al., 2023) and inflammation (Bouchet et al., 2023). Therefore, we investigated the effects of acute unavoidable stress (Paré and Glavin, 1986; Glavin et al., 1994; Zimprich et al., 2014) on feed-forward inhibition in the hippocampal-EC loop mediated by CB1-INs. Mice were restrained for 1 h prior to decapitation. First, we compared the levels of sustained suppression of GABA release at CB1-IN to LVb cell synapses in naïve and stressed animals. This was achieved by measuring the effects of CB1-R antagonist application on the amplitude of IPSCs recorded from connected pairs of neurons. In slices from naïve mice AM251 caused small, but significant enhancement of IPSC amplitudes (Supplementary Figure 1B). However, in pairs recorded from stressed animals, CB1R antagonist administration resulted in significantly stronger amplification of IPSCs (Supplementary Figure 1B), suggesting that acute stress leads to long lasting activation of CB1Rs. Thus, stress induced elevation of endocannabinoid levels may facilitate SPW-R driven excitation of LV pyramidal cells in mEC via selective suppression of CB1-IN mediated feed-forward inhibition. To test this hypothesis, we compared the amplitudes and halfwidth of compound SPW-R driven PSPs before and after AM251 application in slices from stressed and naïve mice. In LVa and LVb cells of naive animals where we expected minimal modulation of CB1-IN IPSPs by tissue endocannabinoids, drug administration didn’t produce any significant alteration of PSP amplitude and duration (Figures 4A, C). However, in stressed animals, CB1R antagonist application strongly reduced the amplitude and halfwidth of compound SPW-R driven responses, suggesting that relief from endocannabinoid block enhanced the IPSP component (Figures 4B, D). In similar experiments performed with depolarizing IPSPs (high Cl^–^ internal solution) AM251 application in stressed animals led to enhancement of PSP amplitude without significant alteration of duration, proving that block of CB1Rs affects SPW-R driven inhibition (Supplementary Figure 6).

## Discussion

In this paper we describe, for the first time, the role of CB1 receptor expressing interneurons located in layer V of entorhinal cortex (EC) in control of excitation flow within the hippocampal-entorhinal loop and show that these interneurons can be modulated by stress. We describe the basic and synaptic properties of Layer V EC CB1-INs. Similarly to layer V EC parvalbumin positive fast spiking interneurons, CB1-INs receive direct excitatory drive from the hippocampus during the hippocampal rhythmic activity, sharp wave-ripple oscillations (Rozov et al., 2020). However, we didn’t find any evidence of the existence of excitatory connections from local Va and Vb, pyramidal to layer V CB1-INs. The domination of distal, hippocampal excitation over local excitatory inputs strongly suggests involvement of CB1-INs in hippocampal-driven feed-forward inhibition. This assumption is further supported by the very high connectivity rates (>80%) and the strength of the GABAergic connection from presynaptic CB1-INs to Va and Vb pyramidal cells. Comparing the synaptic interaction of either CB1-INs or FS-INs with the surrounding layer V pyramidal neurons, we found that both CB1-IN connectivity and the efficacy of inhibition at their synapses were significantly higher than for connections formed by FS-INs. Thus, summarizing the existing data on layer V interneurons we can conclude that CB1-INs are substantially involved in hippocampus-driven feed-forward “GABAergic control” of layer V entorhinal cortex circuitry, and they do not contribute much to local feed-back inhibition.

Expression of CB1 receptors equips the feed-forward inhibitory chain made by these interneurons with activity sensitive gain control. Endocannabinoid modulation of GABAergic synapses can occur at the several levels and different time scales. The first level is DSI-like reduction of CB1-IN-mediated inhibition at given synapses. Enhanced subthreshold activity in the target postsynaptic Va and Vb pyramidal neurons could be translated into temporal, on a tenths of seconds timescale, endocannabinoid-dependent suppression of CB1-IN-mediated inhibition. Selective reduction of inhibition of those layer V pyramidal cells that receive stronger hippocampal excitatory drive may contribute to the formation of neuronal engrams similar to that observed in the hippocampus and other brain regions (Kim et al., 2013; Morrison et al., 2016; Stefanelli et al., 2016; Han et al., 2022). Interestingly, rearrangement of CB1-IN- and FS-IN-mediated inhibition has been shown for hippocampal CA1 pyramidal cells which were “engaged into new object memory formation.” Perisomatic inhibition of *Fos*-expressing CA1 pyramidal neurons by local parvalbumin expressing FS-INs was enhanced, while perisomatic inhibition by hippocampal CB1-INs was reduced (Yap et al., 2021). The possibility of similar long lasting tuning of efficacy at layer V CB1-INs synapses triggered by stimuli that can lead to formation of new engrams should be addressed by combining behavioral, molecular and electrophysiological experiments.

The second way that endocannabinoids may influence the impact of CB1-IN mediated inhibition is more global and arises from glucocorticoid modulation of endocannabinoid production. It is known that the concentration of circulating 2-arachidonoyl glycerol increases in various brain regions including the hippocampus in response to glucocorticoid administration or as a result or acute, restraint, stress (Hill and McEwen, 2010; Morena et al., 2016; Balsevich et al., 2017). CB1R-mediated components of the stress response and stress adaptation have also been observed at the behavioral level (Atsak et al., 2012; Santori et al., 2020). In this study we tested whether 1 h of restraint stress can influence the weight of CB1-INs in the net SPW-R driven inhibition of layer V EC pyramidal cells. Indeed, while in naïve animals application of a CB1R antagonist did not have any effect on the amplitude and halfwidth of triggered PSPs in Va and Vb pyramidal neurons, in slices from the brains of stressed animals CB1R blockade caused significant reduction of both the amplitude and duration of SPW-R driven responses. The effects of AM251 on amplitude and kinetics were also observed in cells recorded with a “high Cl^–^“ internal solution, that excludes possible effects of CB1R occlusion on the excitatory component of SPW-R triggered PSPs. These data suggest that acute stress results in sustained activation of CB1Rs at GABAergic synapses onto Va and Vb neurons, that greatly reduces net inhibition, which in turn promotes the efficiency of hippocampus-driven excitation to the deep layers of EC. Hence, depending on the mechanism, endocannabinoid production CB1-INs can either “allow” preferential information flow from the hippocampus to the most active receiving excitatory neurons in layer V EC or rapidly reduce inhibitory control of the entire population of deep layer pyramidal cells. As a future extension of this study, it would be interesting to evaluate the role of CB1-INs and endocannabinoid signaling in controlling the generation and propagation of epileptiform activity.

Thus, this study provides valuable and novel information about: (i) integration of CB1-INs into the local network in layer V of the entorhinal cortex; (ii) the organization of hippocampal inhibitory control over information processing in the entorhinal cortex and (iii) the cellular mechanisms for translating environmental stress to neuronal activity.

## Materials and methods

### Preparation of mouse brain slices

Horizontal brain slices (450 μm thick) containing the hippocampus and entorhinal cortex were obtained from male C57BL/6N mice or genetically modified reporter mice B6.Cg-Gad1^*TM*1*Tama*^ (GAD67-GFP) 10–12 weeks of age using a standard procedure (Roth et al., 2016). All experimental protocols were approved by the State Government of Baden-Württemberg (Projects T100/15 and G188/15) or by the Local Ethical Committee of Kazan Federal University (#24/22.09.2020). Mice were killed under deep CO_2_-induced anesthesia. After decapitation, brains were rapidly removed and placed in cold (1–4°C) oxygenated artificial CSF (ACSF) containing the following (in mM): 124 NaCl, 3 KCl, 1.6 CaCl_2_, 1.8 MgSO_4_, 10 glucose, 1.25 NaH_2_PO_4_, and 26 NaHCO_3_, saturated with carbogen (95% O_2_ and 5% CO_2_), with pH 7.4 at 34°C. Horizontal brain slices containing the intermediate/ventral portion of the hippocampus and connected areas of the entorhinal cortex were cut using a vibratome slicer (VT1200S, Leica). Section level was between approximately -3.7 and -5 mm along the dorsoventral axis. To better preserve connectivity between the hippocampus and entorhinal cortex, slices were cut with an angle of ∼15° toward the ventral side. Before electrophysiological recordings, slices were allowed to recover for at least 2 h. Slices that were used for the registration of oscillatory activity were transferred into a Haas-type interface chamber (Haas et al., 1979), and superfused with ACSF at a rate of 1.5–2 ml/min at 34 ± 1°C. Otherwise, slices were stored in a submerged incubation chamber at room temperature.

### Examining connectivity of ec layer V CB1-INs and their sensitivity to endocannabinoids

Dual whole-cell recordings were performed at 32 ± 1°C. Slices were continuously superfused with an extracellular solution containing the following (in mM): 125 NaCl, 2.5 KCl, 25 glucose, 25 NaHCO_3_, 1.25 NaH_2_PO_4_, 2 CaCl_2_, and 1 MgCl_2_, bubbled with 95% O_2_/5% CO_2_. The pipette solution contained the following (in mM): 110 K-gluconate, 30 KCl, 8 NaCl, 10 HEPES, 4 Mg-ATP, 0.3 Na-GTP, and 10 Na_2_-phosphocreatine, adjusted to pH 7.3 with KOH. To study synaptic connections, presynaptic cells were stimulated with a 10 Hz train of five suprathreshold current pulses, which were repeated every 10 s. All paired recordings used for connectivity analysis were conducted in CC mode. During recordings, cells were held at resting membrane potential. Averages of 50–100 consecutive sweeps were used for the analysis of postsynaptic responses. Depolarization induced suppression of inhibition at synapses formed by CB1-INs were tested in a separate set of experiments where postsynaptic Va and Vb pyramidal neurons were dialyzed with a Cs^+^-based “high Cl^–^“ internal solution. DSI was induced by depolarization of the postsynaptic cell to 0 mV for 2 s.

The effect of acute stress on the magnitude of endocannabinoid-dependent chronic suppression of inhibition at synapses formed by CB1-INs was assessed by comparing the enhancement of IPSC amplitudes upon application the CB1R antagonist AM251 (2 mM). Postsynaptic cells were recorded with a Cs^+^-based “high Cl^–^“ internal solution and held at −70 mV. The effect of AM251 was measured 15 min after drug application.

### Simultaneous recordings of SPW-Rs and postsynaptic responses from LV neurons

After resting in an interface chamber, slices were transferred into a modified double perfusion submerged chamber (Hájos and Mody, 2009) and perfused with ACSF at a rate of 9–10 ml/min at 32 ± 1°C. Extracellular local field potentials (FPs) were recorded from stratum pyramidale of hippocampal area CA1 using ACSF-filled borosilicate glass electrodes with a tip diameter of 3–5 μm. Following this protocol, submerged hippocampal slices showed spontaneously occurring SPW-Rs (Maier et al., 2003), which could be reliably observed for at least 2 h. Extracellular FPs were amplified 100 × with an EXT 10-2F amplifier (npi electronics). Signals were digitized at 10 kHz with an analog-to-digital converter [ADC; MICRO 1401 mkII, Cambridge Electronic Design (CED)] and saved on a computer using PATCHMASTER software (HEKA) for offline analysis. Patch-clamp recordings were performed using two EPC7 amplifiers (HEKA). Layer V neurons (Va and Vb excitatory cells, FS-INs and CB1-INs) were identified by their location in the slice, the appearance of the cell body on the IR-image and characteristic firing pattern.

Whole-cell current-clamp (CC) recordings were performed using borosilicate glass pipettes with resistances of 5–7 MΩ containing depending on experimental needs either “low Cl^–^” or “High Cl^–^” intracellular solutions. The low Cl^–^ solution consisted of (in mM): 144 K-gluconate, 4 KCl, 10 HEPES, 4 Mg-ATP, 0.3 Na-GTP, and 10 Na_2_-phosphocreatine, adjusted to pH 7.3 with KOH. The high Cl^–^ solution consisted of (in mM): 110 K-gluconate, 30 KCl, 8 NaCl, 10 HEPES, 4 Mg-ATP, 0.3 Na-GTP, and 10 Na_2_-phosphocreatine, adjusted to pH 7.3 with KOH. During recordings, cells were held at resting membrane potential, unless otherwise indicated.

For recordings of postsynaptic currents patch pipettes were filled with a Cs^+^-based “low Cl^–^“or “High Cl^–^“ internal solution. In the Cs^+^-based internal solutions K^+^ was substituted with an equimolar concentration of Cs^+^. In Voltage-clamp experiments holding membrane potentials were corrected for the liquid junction potential of approximately -15 mV.

In the experiments studying the effects of depolarization on the inhibitory components of SPW-R-driven postsynaptic currents, the postsynaptic neurons were depolarized to 0 mV for 5 s. The postsynaptic pyramidal cells in this case were recorded with Cs^+^-based internal solutions. To study the effect of postsynaptic high frequency firing on the inhibitory components of SPW-R-driven PSPs, neurons dialyzed with K^+^-based internal solutions were injected with 5 s depolarizing current pulses (300–350 pA). The concentration of Cl^–^ in the intracellular solution was determined by experimental needs and is stated in the main text.

### Stress protocol

Animals of the stress group were restrained in well-ventilated 50 ml tubes and left undisturbed a separate room from the other animals for 1 h (Zimprich et al., 2014). After the restraint period the mice were sacrificed, then brains were dissected and sliced as described above.

### Data analysis

Raw data were digitally filtered using the RC (resistor–capacitor) filter routine of MATLAB [bandpass: 1–80 Hz for SPW-Rs; 1–500 Hz for postsynaptic potentials (PSPs); and 0.1–500 Hz for postsynaptic currents (PSCs)]. For signal detection, a two-threshold method was applied as follows. First, events exceeding three SDs of the most silent 10-s period of the full-length recording were considered as SPW-Rs, PSPs, or PSCs, respectively. Second, approximate onsets and offsets of the SPW-R events were defined as times when the signal intersected a threshold of 1 SDs of the most silent 10-s period. Exact SPW-R onset was defined as the time when the first derivative of the FP (low-pass filtered at 40 Hz) reached a threshold of 0.02 mV/ms. For PSP detection, approximate onsets and offsets of the signals were defined as the time when the signal intersected a threshold of 1 SD. Exact PSP onset was defined as the time when the first signal derivative (low-pass filtered 500 Hz) reached a threshold of 0.1 mV/ms. For PSCs, approximate onsets and offsets were defined as times when the signal intersected a threshold of 0.5 SDs. Exact PSC onset was defined as the time when the first signal derivative (low-pass filtered at 500 Hz) reached a threshold of 10 pA/ms. The correlation between SPW-Rs and PSPs or PSCs was calculated based on cross-correlograms of onsets. Event amplitudes were estimated as the maximum value between onset and approximate offset with subtraction of baseline level (median value from a 3 ms window before onset). Event half-width was estimated as the duration at the half-amplitude level. Latencies between SPW-Rs in CA1 and PSPs/PSCs in the mEC were defined as the time interval between onset of field-SPW-R and onset of postsynaptic events. PSPs or PSCs in the mEC were considered SPW-R driven if their onset time was < 50 ms following the beginning of an SPW-R event in CA1.

For ripple-associated PSPs, the first derivative of potential was calculated to estimate the contribution from excitatory and inhibitory current components (e-PSP and i-PSP). The corresponding amplitudes were calculated as the maximum and minimum peak values of the first derivative of potential during PSP (Supplementary Figure 5).

All data were analyzed offline using PatchMaster (HEKA), SigmaPlot (Systat) and MATLAB R2012 (MathWorks). Values of EPSP/PSP amplitudes of connected pairs were calculated from averaged first synaptic responses in trains of 5.

### Statistical analysis

Quantitative data from multiple slices are given as the median (P_25_; P_75_). Data in figures are presented as medians (P_25_; P_75_) and individual values. Whiskers show minimum and maximum values. Statistical analysis was performed using SigmaPlot (Systat), GraphPad (InStat, GraphPad Software) or Matlab Statistics Toolbox. Mann–Whitney *U test*, Wilcoxon Rank Sum test or Fisher’s exact test were used for statistical comparisons as indicated in the text. A *p* value < 0.05 was regarded as significant (for all data: *p < 0.05, ***p < 0.001, ns, not significant).

## Data availability statement

The raw data supporting the conclusions of this article will be made available by the authors, without undue reservation.

## Ethics statement

The animal study was approved by the State Government of Baden-Württemberg (Projects T100/15 and G188/15) and the Local Ethical Committee of Kazan Federal University (#24/22.09.2020). The study was conducted in accordance with the local legislation and institutional requirements.

## Author contributions

AN: Investigation, Formal analysis, Writing—review and editing. DJ: Investigation, Writing—original draft, Validation. AV: Investigation, Writing—original draft. FV-R: Investigation, Writing—original draft. AR: Funding acquisition, Investigation, Supervision, Writing—original draft.
