# Supplementary material for: Acute stress modulates hippocampal to entorhinal cortex communication
Source: Front Cell Neurosci. 2023 Dec 7;17:1327909. doi: 10.3389/fncel.2023.1327909 (PMC10740169; doi:10.3389/fncel.2023.1327909)
Supplement: Supplementary file 1 [file Image_1.pdf]

Supplementary figures

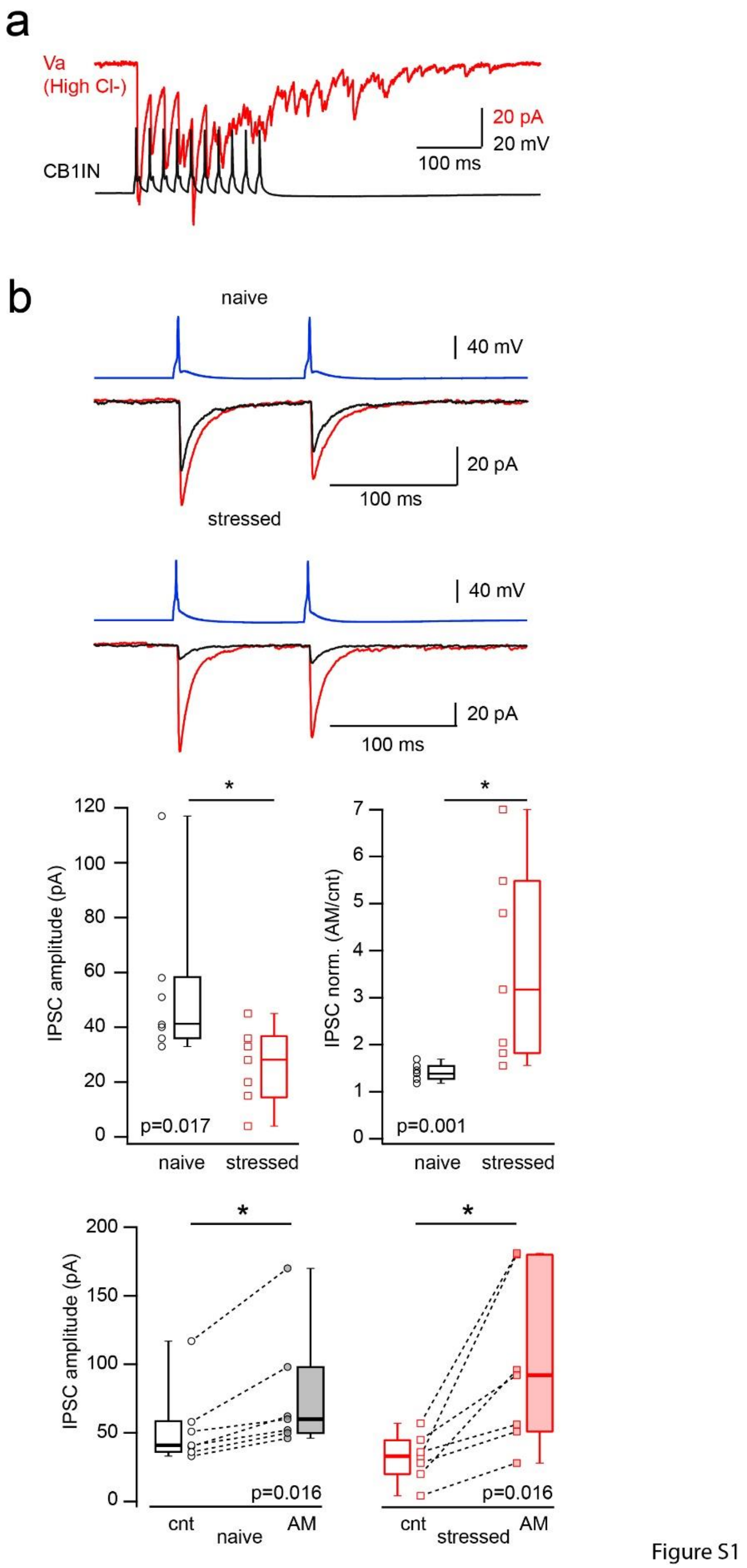

Figure S1. Properties of mEC CB1IN to LV pyramidal cell synapses.

a. High frequency stimulation (10 APs; 50 Hz) of mEC CB1INs triggers profound, long-lasting asynchronous GABA release. Current clamp recording

of presynaptic mEC CB1IN in black trace, and voltage clamp recording of post synaptic pyramidal cell in red trace.

b. Acute stress enhances chronic endocannabinoid suppression of GABA release at mEC CB1-IN to LV pyramidal cell synapses. Averaged traces show presynaptic APs in CB1INs (blue) and averaged postsynaptic IPSCs before (black) and after (red) CB1R antagonist (AM251; 2 μM) application recorded in slices from naïve and stressed mice. The left box plot compares averaged IPSC amplitudes recorded before AM251 application in naïve (black) and stressed (red) animals. The right plot compares the effects of CB1R blockade on synaptic efficacy. Symbols show data from individual neurons (n=7 in each group) The box plot on the bottom demonstrates changes of IPSC amplitudes in individual experiments in control and stressed animals. Data on the box plots are presented as the median (P25; P75). The significance of the differences was assessed by Mann-Whitney test (* $p<0.05$)

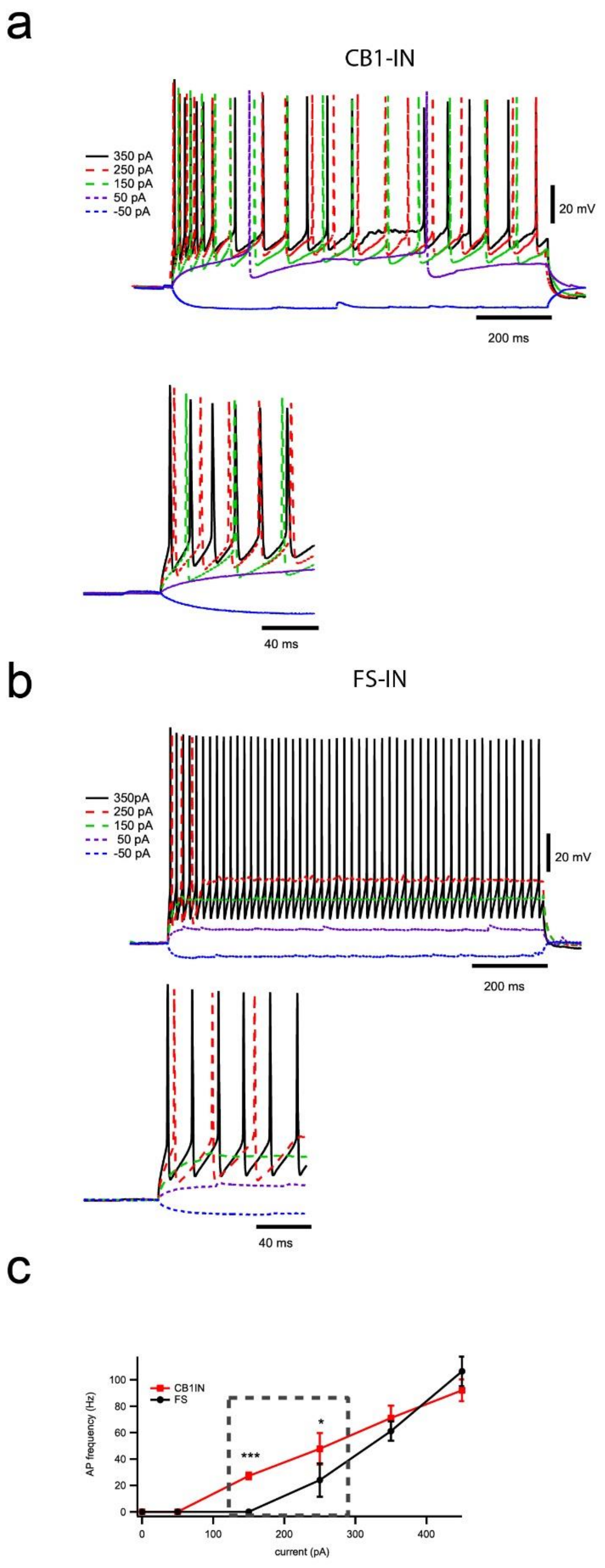

Figure S2.

Figure S2. Excitability of mEC CB1-INs and FS-INs.

Voltage responses triggered by different amplitude current injections in CB1-IN (a) and FS-IN (b). c. AP firing frequency measured during the first 100 ms of depolarization and plotted as the function of injected current amplitude.

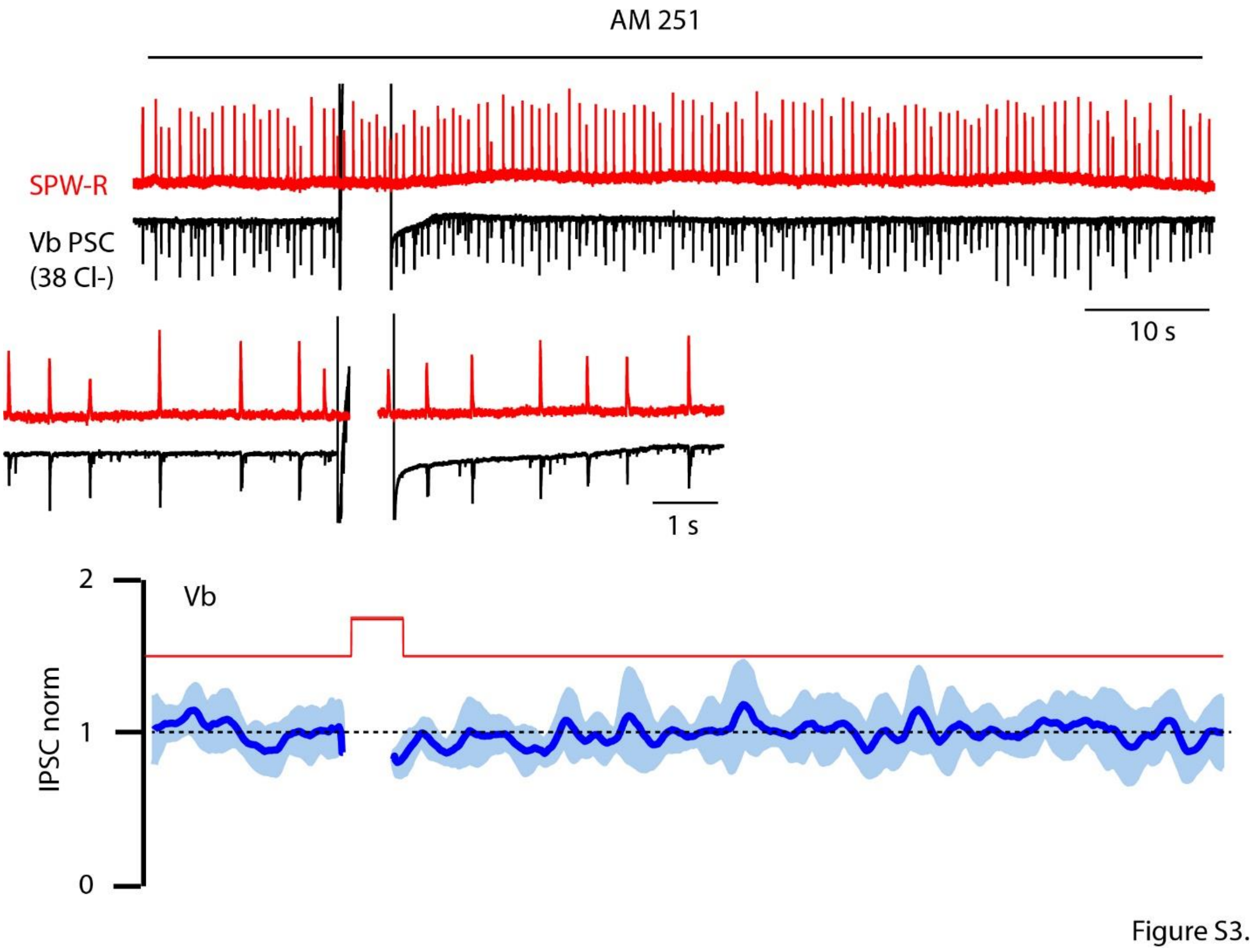

Figure S3. CB1R antagonist occludes DSI of SPW-R triggered IPSCs

Traces represent spontaneous SPW-Rs (red) in CA1 and associated PSC (V_h -90 mV) in a mEC Vb neuron recorded with high-Cl^- (38 mM) internal solution in the presence of AM251. Depolarization of the postsynaptic cell did not have any effect on PSC amplitudes. Plots underneath shows averaged time course (mean ± SEM) PSC efficacy before and after depolarization (n = 5; $p>0.05$). For statistical analysis the PSC amplitudes within the 5 second windows prior to depolarization and right after depolarization were compared.

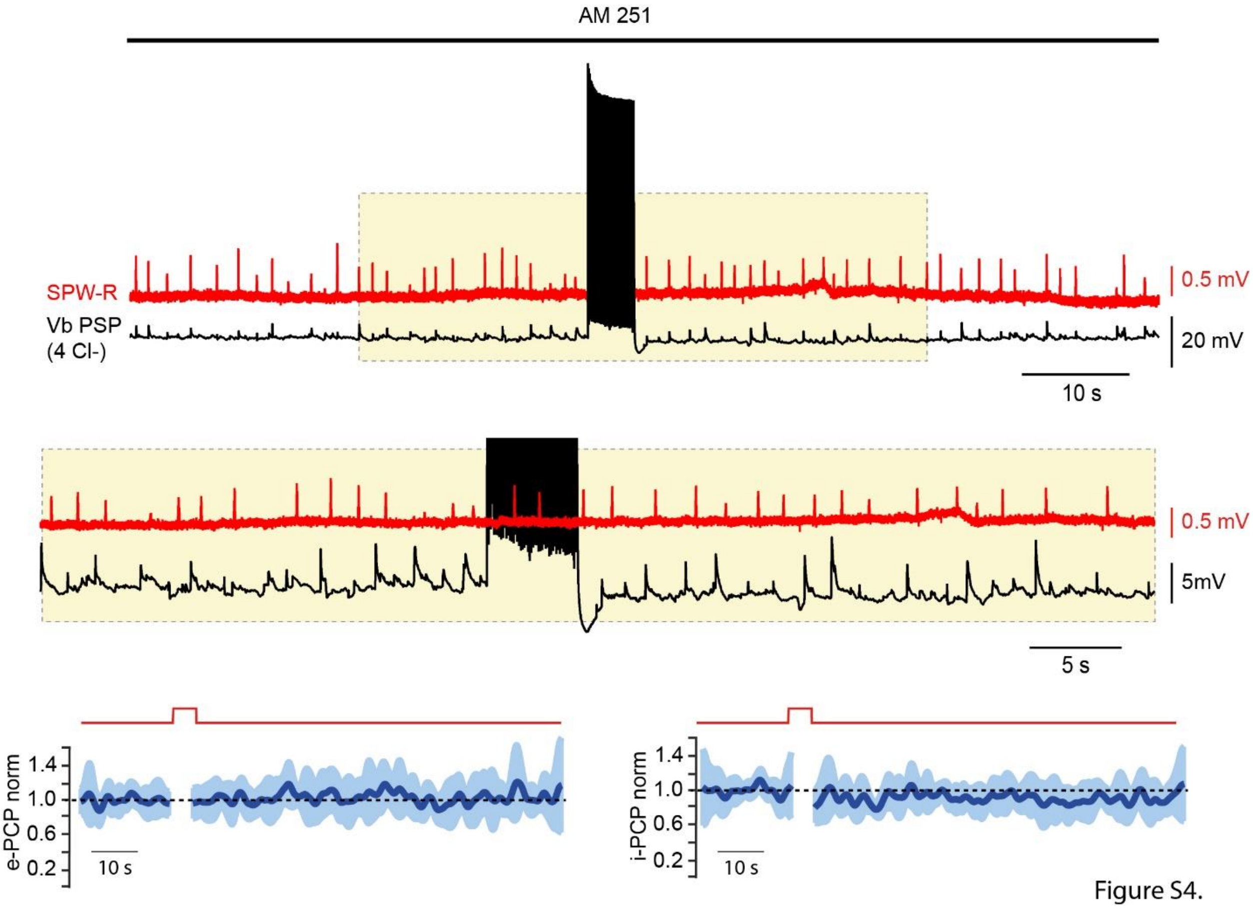

Figure S4. CB1R antagonist occludes DSI of SPW-R triggered IPSPs

Traces represent spontaneous SPW-Rs (red) in CA1 and associated PSPs (black; Vm -65 mV) in mEC LVb (n=8) recorded with a low-Cl^- internal solution in the presence of AM 251. A train (5 s) of high frequency APs in the postsynaptic cell temporally does not affect amplitude and duration of PSPs. Plots show averages of normalized amplitudes of excitatory (e-PSP; $p>0.05$) and inhibitory (i-PSP; $p>0.05$) components of SPW-R driven responses. For statistical analysis the e-PSP and i-PSP amplitudes within the 5 second windows prior to depolarization and right after depolarization were compared.

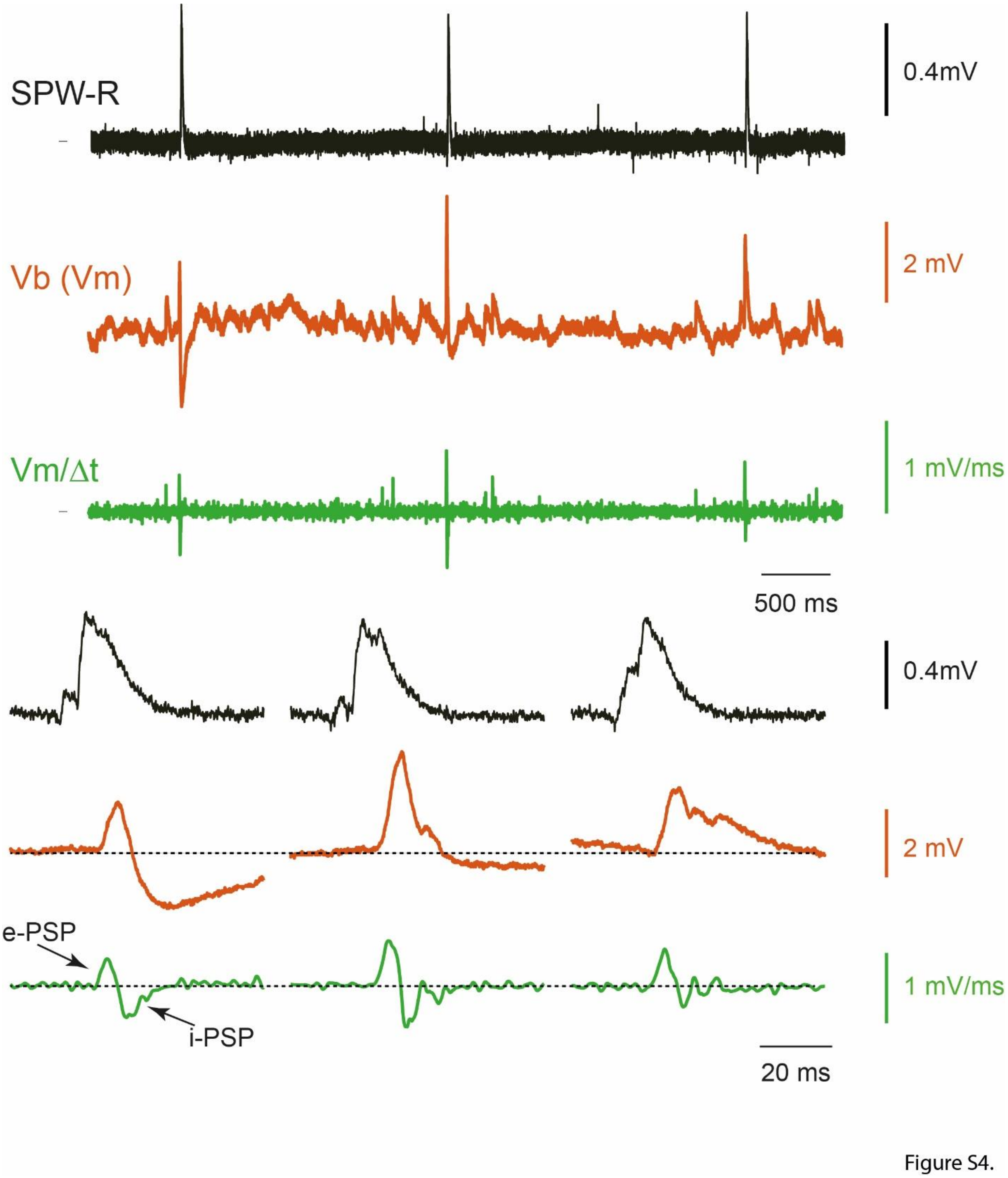

Figure S5. Compound SPW-R driven PSPs consist of excitatory and inhibitory components.

Example traces of SPW-R (black), dual component PSPs in a Vb pyramidal cell (red) and the corresponding fist derivative trace with excitatory (e-PSP) and inhibitory (i-PSP) components (green).

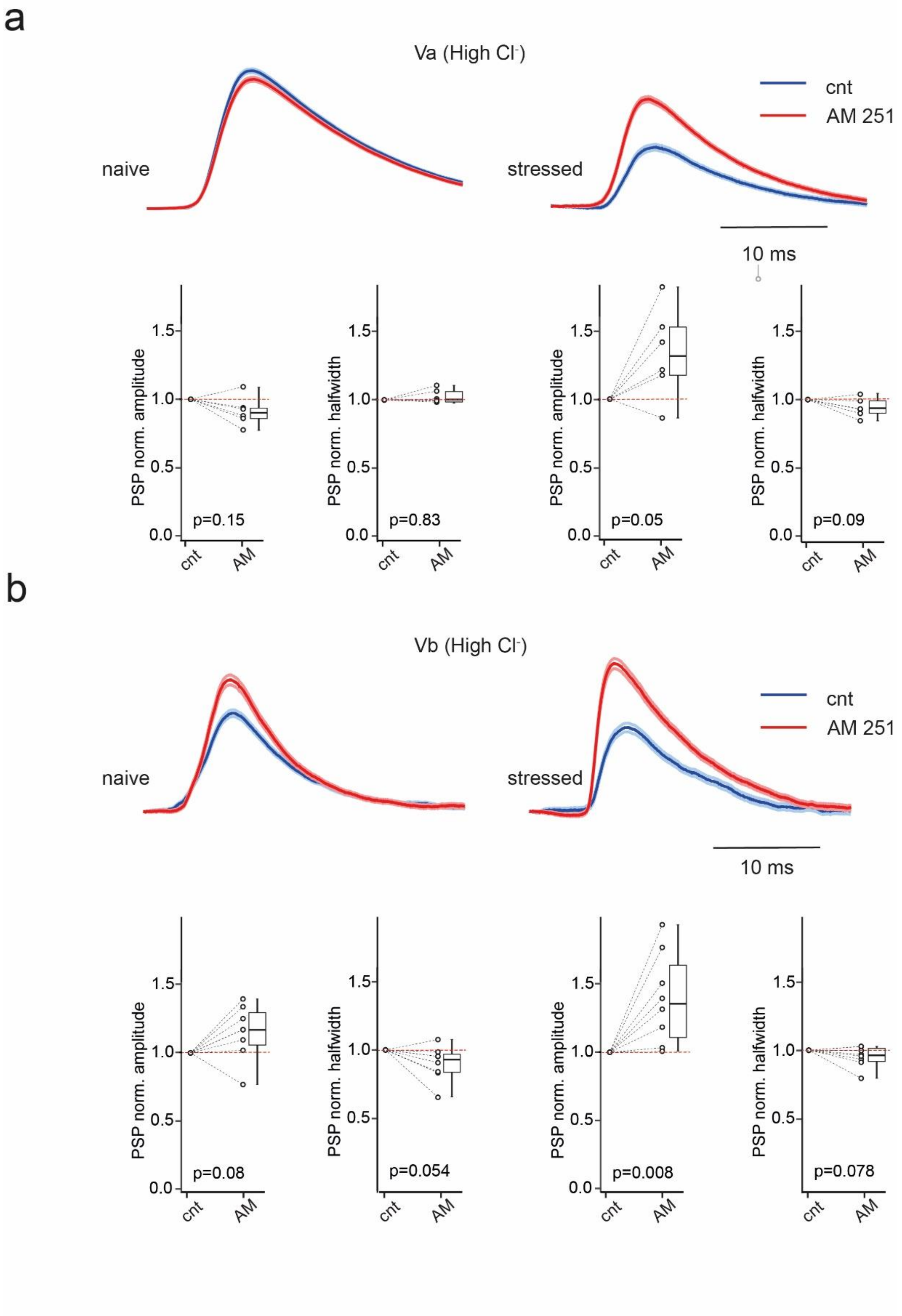

Figure S5.

Figure S6. Effect of CB1R blockade on the amplitude and duration of PSPs in cells dialyzed with high-Cl^- internal solution in slices from naïve and stressed mice.

a. Plots show normalized (AM251/control) amplitude (left) and halfwidth (right) values in individual Va neurons and pooled data (presented as the median;

P25; P75) obtained from control mice (left plots; n= 6) and stressed animals (right plots; n=6) pyramidal cells.

b. The same as in (a) recorded from Vb neurons in the brain slices of control (n=7) and stressed mice (n=8). Note that in both types of pyramidal neurons blockade of CB1R results in significant enhancement of PSP amplitude in slices obtained from the stressed mice. The significance of the differences was assessed by Wilcoxon Signed Rank Test, p-values are indicated on the plots.
